# G2H: A Precise Block-Scanning Strategy for Genetic Background Assessment in Maize Backcross Breeding

**DOI:** 10.3390/genes16121480

**Published:** 2025-12-10

**Authors:** Xiangyu Qing, Weiwei Wang, Liwen Xu, Yunlong Zhang, Yikun Zhao, Jianrong Ge, Xuelei Shen, Rui Wang, Yingjie Xue, Fengge Wang

**Affiliations:** 1 Maize Research Institute, Beijing Academy of Agriculture and Forestry Sciences, Key Laboratory of Crop DNA Fingerprinting Innovation and Utilization (Co-Construction by Ministry and Province), Beijing Key Laboratory of Maize DNA Fingerprinting and Molecular Breeding, Beijing 100097, China; 15661204773@163.com (X.Q.); vivianwang8502@163.com (W.W.); xulw0408@126.com (L.X.); zhangyunlongfrank@163.com (Y.Z.); zhaoqiankaisteam@126.com (Y.Z.); 13439992517@163.com (J.G.); shenxuelei2018@163.com (X.S.); wangrui@baafs.net.cn (R.W.); yingjiexue_zea@126.com (Y.X.); 2 National Key Laboratory of Crop Genetic Improvement, Huazhong Agricultural University, Wuhan 430070, China

**Keywords:** genetic background recovery, panoramic assessment, backcross breeding, haplotype, maize

## Abstract

(1) Background: Backcross (BC) breeding is a key technology of crop improvement, yet its efficiency largely depends on the precise assessment of the genetic background recovery. Conventional molecular marker-assisted techniques suffer from inadequate genomic coverage or an inability to resolve true chromosomal structure. (2) Methods: To address major issues in maize BC breeding, we devised a G2H block-scanning strategy. This approach converts high-density point markers into haplotype blocks, enabling precise evaluation of the genetic background in backcross progenies. A key innovation is the CFDI, which quantifies the distribution of unrecovered fragments, allowing for visual tracking of chromosomal recombination and identification of ideal individuals with both a high genetic background recovery rate and few small fragments retention. (3) Results: We validated the accuracy and effectiveness of the G2H strategy across multiple backcross generations. Through enabling a precise “point-to-line-to-area” panoramic assessment of genetic background, G2H provides a powerful tool for developing ideal breeding materials with pure genetic background and minimized linkage drag. (4) Conclusions: Notably, this strategy significantly shortens the breeding cycle by 2–3 generations compared to conventional background assessment methods, thereby accelerating precision molecular design breeding in crops.

## 1. Introduction

Maize (*Zea mays* L.), as a globally significant food, feed, and energy crop, plays a crucial role in ensuring food security and promoting sustainable agricultural development through genetic improvement. Backcross breeding serves as a fundamental strategy in crop genetic improvement, enabling the targeted introgression of desirable alleles (such as those conferring disease resistance or stress tolerance) from a donor parent (DP) into the genetic background of an elite recurrent parent (RP) while largely preserving its agronomic value [1,2]. This approach is particularly valuable in modern breeding programs, where it facilitates the rapid enhancement of established lines through the incorporation of specific traits, often supported by biotechnological tools. However, the efficiency of conventional BC breeding is constrained by its reliance on phenotypic selection and extended generational cycles, typically requiring 6–8 or more generations to adequately recover the RP genetic background, thereby incurring substantial time and resource costs [3,4].

The integration of molecular marker-assisted selection (MAS) has markedly improved the precision of BC breeding. Early MAS efforts largely utilized simple sequence repeat (SSR) markers, yet their limited genomic density and technical inconsistencies restricted their utility in genome-wide background selection [5]. The advent of single-nucleotide polymorphism (SNP) markers offered higher throughput and genome-wide coverage, significantly accelerating background recovery [6,7]. Nevertheless, SNP-based methods still operate at the level of discrete point-based data, which limits their ability to accurately delineate recombination breakpoints or characterize chromosomal segment structures [8].

Recent advances in genotyping and haplotyping technologies have led to the development of haplotype-based marker systems, such as haplotype tag polymorphism (HTP). By aggregating linked SNPs into continuous chromosomal blocks, HTP markers provide a more biologically meaningful representation of genomic inheritance, improve resolution in tracking recombination events, and enhance the accuracy of background recovery estimation [8,9]. Despite these improvements, critical methodological gaps remain. Existing HTP frameworks often lack standardized and robust protocols for block assignment and fragment state quantification. Moreover, they offer limited capacity to evaluate the distribution and complexity of residual donor fragments—a significant oversight since numerous small, scattered segments hinder efficient background recovery in advanced generations [10].

To address these shortcomings, this study was designed to develop and validate a novel G2H block-scanning strategy for maize BC breeding. The primary objectives were as follows:

(i) To establish a standardized system, which divides the maize genome into 6163 seamless HTP blocks, covering 3587 inbred line resources, laying the foundation for high-coverage genome-wide analysis [11];

(ii) To introduce a new index for quantifying the size and distribution of unrecovered donor fragments, enabling the selection of individuals with optimal chromosomal structural features;

(iii) To validate the utility of this integrated approach in tracking background recovery dynamics and identifying ideal candidates with high genetic purity and minimal linkage drag across successive BC generations.

## 2. Materials and Methods

### 2.1. Test Materials

Test materials were selected from the Q1 population (Q1B1 denotes the BC_1_ generation of this population, Q1B2 denotes the BC_2_ generation). High-quality maize inbred lines served as RP, while donor inbred lines carrying the target trait functioned as DP or were used to construct chromosome substitution populations. A BC_1_ population was constructed with a population size ≥ 200 plants (determined based on statistical power calculations). Control materials included the RP, DP, and F_1_ hybrid (RP × DP). Population samples were screened using forward markers and 10 KASP markers. Forty-nine individual plants selected from the Q1B1 population and 33 individual plants selected from the Q1B2 population underwent genetic background scanning using the Maize6H-60K chip [12].

### 2.2. Genotyping and Loci Filtering

DNA was extracted from maize population leaves using the SDS method for individual plants [13]. Genotyping of the DP, RP, and BC population was performed on the Gene Titan platform using the high-quality Maize6H-60K (Thermo Fisher Scientific Affymetrix, Waltham, MA, USA) [12]. Raw CEL files were analyzed using Axiom Analysis Suite software (V4.0.3.3) following best practices [12]. Quality control parameters were set as: DQC > 0.82 and QC call rate > 95% to obtain high-quality genotype data.

### 2.3. Construction and Application of the G2H System

#### 2.3.1. Parental Polymorphic Loci

Parental polymorphism loci, defined as loci where both the DP and RP are homozygous but possess different genotypes, were identified and retained for subsequent analysis. All other loci were discarded.

#### 2.3.2. Genotype Comparison and Digital Coding

To assess genetic background recovery, each locus in the BC population was compared individually with the RP. A locus was coded as “1” if the BC individual was homozygous and matched the RP genotype, indicating recurrent status. A heterozygous genotype consistent with the parental alleles was coded as “2”. All other cases were treated as missing data and recorded as “0”. The proportion of recurrent parent genome (PRPG) based on SNP markers was calculated as follows:

PRPG = A/B × 100%

where A denotes the number of SNP loci identical to the RP genotype, and B represents the total number of polymorphic SNPs between the two parents with missing SNP loci excluded.

#### 2.3.3. Conversion of Digitally Encoded Genotypes to HTP Blocks

Based on the previously established HTP blocks, the genotype status encoded in numerical format (1 or 2) was converted into block-wise haplotype information. For each HTP block, the number and proportion of recurrent (code 1) and non-recurrent (code 2) polymorphic SNP loci were tallied. HTP blocks lacking polymorphic SNPs were classified as missing and assigned a value of 0.

To determine the recovery status of informative HTP blocks, a threshold for the proportion of recurrent SNPs within each block was established through a systematic calibration procedure. This involved identifying two types of chromosomal extremes in BC individuals: chromosomes with recovery rates > 95% and scatteredly distributed unrecovered SNPs (classified as recovered), and those with recovery rates ≤ 10% with scatteredly distributed recovered SNPs (classified as unrecovered). The distribution of SNP recovery proportions within HTPs from these chromosomes was analyzed to define an objective threshold.

An HTP block was classified as recovered (coded as 1) if its proportion of recurrent SNPs exceeded the threshold and as unrecovered (coded as 2) otherwise. The HTP-based recurrent parent background recovery rate (HTBR) was then calculated as follows:

HTBR = NS_HTP_/N_HTP_

where NS_HTP_ denotes the number of HTP segments consistent with the RP genotype; N_HTP_ represents the total number of non-missing HTPs in the individual.

#### 2.3.4. HTP Smoothing

To ensure chromosome data integrity, HTP blocks monomorphic between parents were assigned a value of 1, indicating RP status. Smoothing was performed using the Circular Binary Segmentation CBS algorithm implemented in the R package PSCBS (version 0.68.0) [14,15]. The function segmentByCBS was executed with the following parameters: significance level alpha = 0.001, undo.SD = 1.5, and min.width = 5. Chromosomal segments were redefined according to the identified breakpoints. For each resulting segment, the raw values of all consistent HTPs were extracted, and the median value was assigned as the final background state, ensuring robust representation of the local genomic background.

#### 2.3.5. Calculation of WGBR and CFDI

The whole-genome background recovery rate (WGBR) was calculated from the smoothed HTP genotype data using the following formula:

WGBR = LS_HTP_/L_HTP_

where LS_HTP_ denotes the total length of HTP segments matching the RP’s genotype, and L_HTP_ represents the total length of HTP segments in the individual. Individuals with WGBR ≥ 95% were classified as high background-reversion (HTBR) plants. Chromosomal recovery patterns were visualized using the package RIdeogram (version 0.2.2) [16].

To quantitatively evaluate the distribution of unrecovered chromosomal fragments, the Chromosomal Fragment Distribution Index (CFDI) was developed. Initially, the number of unrecovered fragments per sample was quantified and standardized (denoted as N*). The calculation process involved the following formula:

N* = (N − N_min_)/(N_max_ − N_min_)

N: Number of unrecovered fragments in the sample;

N_max_: Maximum number of unrecovered fragments across all individual plants;

N_min_: Minimum number of unrecovered fragments across all individual plants.

The proportion of unrecovered fragments per chromosome was calculated for each plant, and the mean P* across all 10 chromosomes was determined. Both the number and size of unrecovered fragments were assigned equal weighting of 0.5. The comprehensive index formula is as follows:

CFDI = (0.5 × N*) + (0.5 × P*)

The CFDI ranges from 0 to 1, with lower values indicating a smaller number of unrecovered fragments and shorter average length.

**Figure 1:**
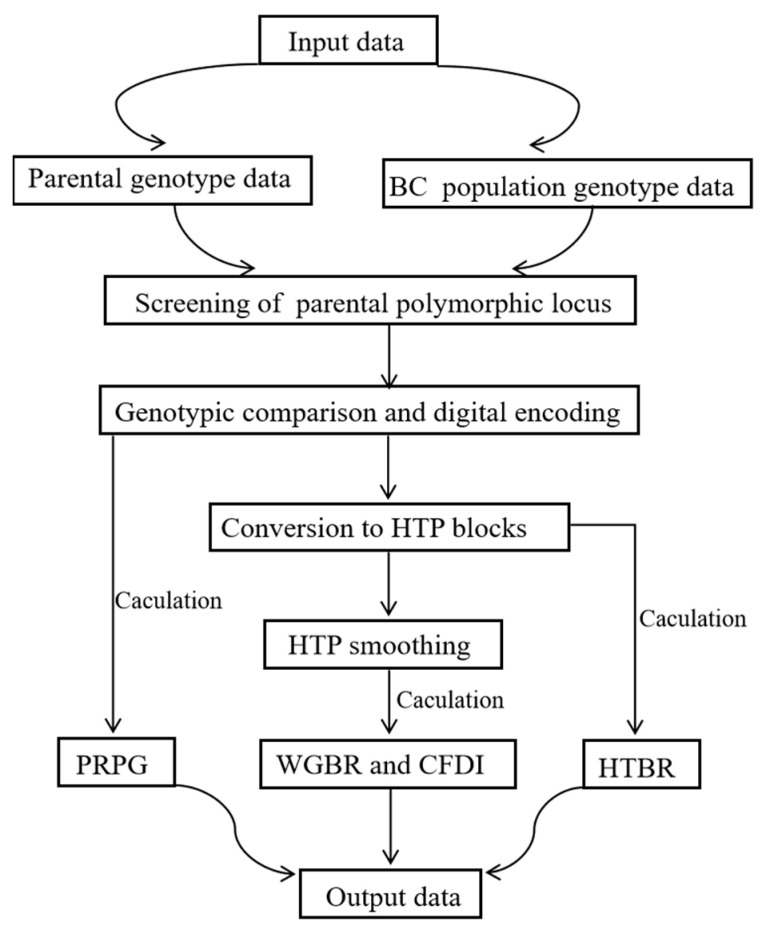
Graphical workflow of the G2H block-based genetic background screening strategy.

The G2H block-based genetic background screening strategy followed here is represented in Figure 1. Python (version 3.9.6) was used for all statistical analyses. All statistical plots were performed using the ggplot2 (version 3.5.1) package in R (version 4.4.1).

## 3. Results

### 3.1. Design of the G2H Block-Based Genetic Background Screening Strategy

**Figure 2:**
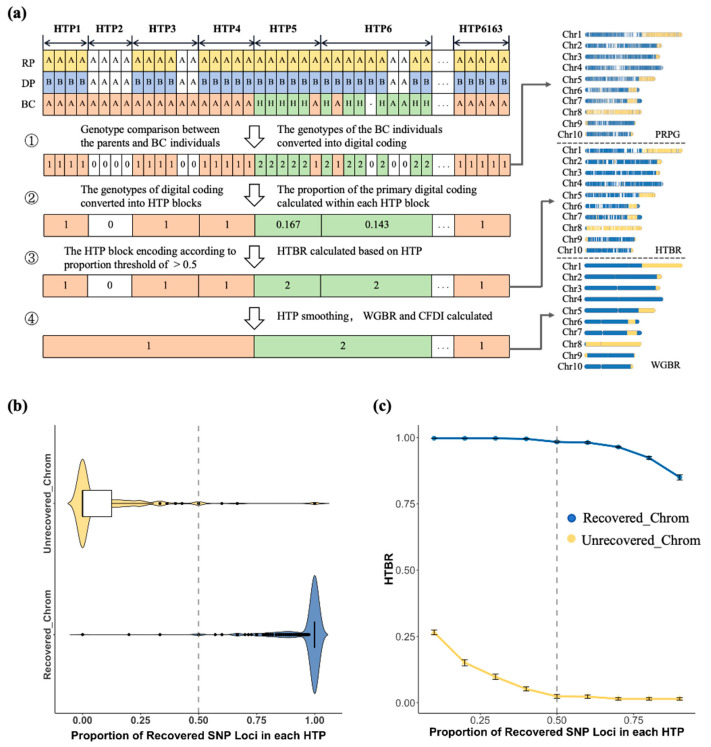
Development of the G2H block-based genetic background screening strategy and threshold analysis: (**a**) Flowchart illustrating the G2H strategy for evaluating the genetic background of BC populations. Genotype categories are color-coded as follows: yellow and blue denote polymorphic loci in parental genotypes; green and orange denote heterozygous or homozygous genotypes matching the RP at that locus; white indicates loci with no parental genotype differences or missing genotype. The overall workflow comprises ① genotype comparison and numerical coding conversion between BC individuals and RP; ② calculation of the proportion of each numerical type within HTP blocks; ③ assignment of haplotype codes to the main body of HTP blocks and calculation of BRR; ④ HTP block smoothing, calculation of genome-wide recovery rates, and comprehensive metrics. (**b**) Distribution of the proportion of recovered SNPs within HTPs in recovered and unrecovered chromosomes. (**c**) The HTP-based recovery rate of recovered and unrecovered chromosomes across different proportion thresholds. Blue and yellow represent recovered and unrecovered chromosomes, respectively.

We developed a novel genetic background scanning technique G2H system, which was designed to enhance the efficiency of maize BC breeding through high-resolution assessment. The G2H framework (Figure 2a) initiates by numerically encoding the genotypes of BC individuals relative to the DP and RP. This method then achieves a critical transformation from discrete genotypes to continuous haplotypes by analyzing the compositional ratios of these numerical codes within predefined HTP blocks. Each block is assigned a definitive haplotype code based on its dominant numerical value, a process that fundamentally enhances the accuracy and interpretability of genetic background evaluation.

Analysis of the distribution of recovered SNP proportions within HTP blocks revealed a clear separation between recovered and unrecovered chromosomes (Figure 2b). In unrecovered chromosomes, 95.1% of HTP blocks showed a recovery proportion below 0.5, whereas in recovered chromosomes, 98.3% of HTP blocks exceeded this value. We further compared the HTP-based recovery rates of these two chromosome types across different proportion thresholds. The results indicated that when the threshold was set to 0.5, the HTP-based recovery rates for both groups stabilized (Figure 2c), supporting the selection of 0.5 as the optimal threshold for determining HTP block recovery status. To improve the consistency of the haplotype map, a digital denoising algorithm was applied to correct inconsistencies and smooth the initial haplotype codes, effectively mitigating biases from individual genotyping errors. Based on the refined haplotypes, the level of genetic background recovery was quantified as the proportion of chromosome length covered by HTP blocks matching the RP haplotype.

Furthermore, the G2H strategy enables precise identification of chromosomal recombination breakpoints. Under the premise that recombination is unlikely within HTP blocks, the boundaries between adjacent blocks with distinct haplotypes were interpreted as potential recombination sites. This capability provides critical insights into the genetic architecture of backcross populations and facilitates the detection of exchange events near target genes. As a result, G2H offers reliable molecular guidance for selecting advanced breeding lines with optimal genetic backgrounds and desired recombination patterns.

### 3.2. Comparative Analysis of G2H Strategy and Traditional Point Marking Methods

**Figure 3:**
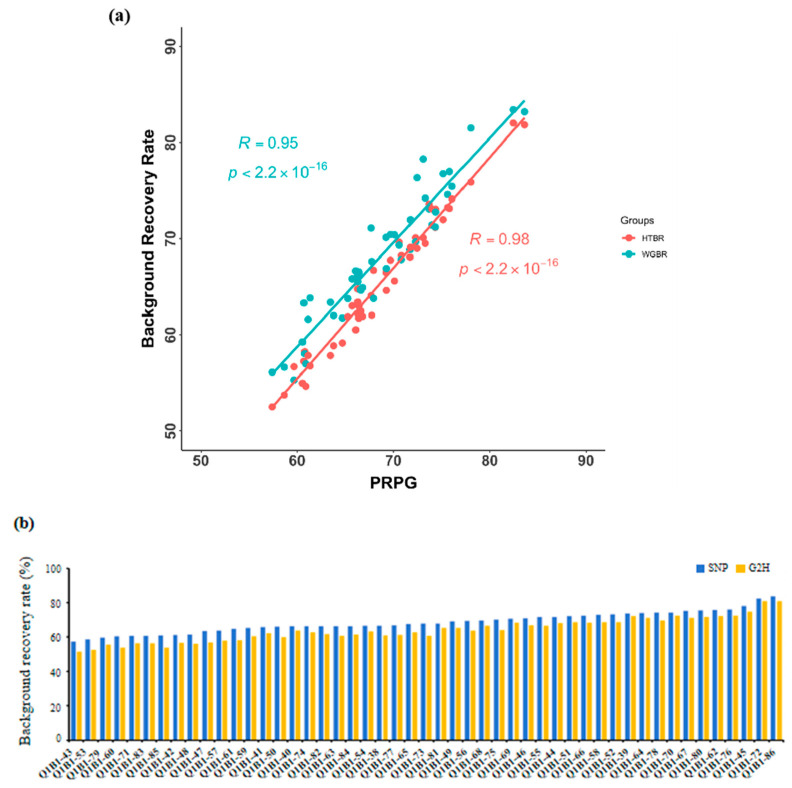

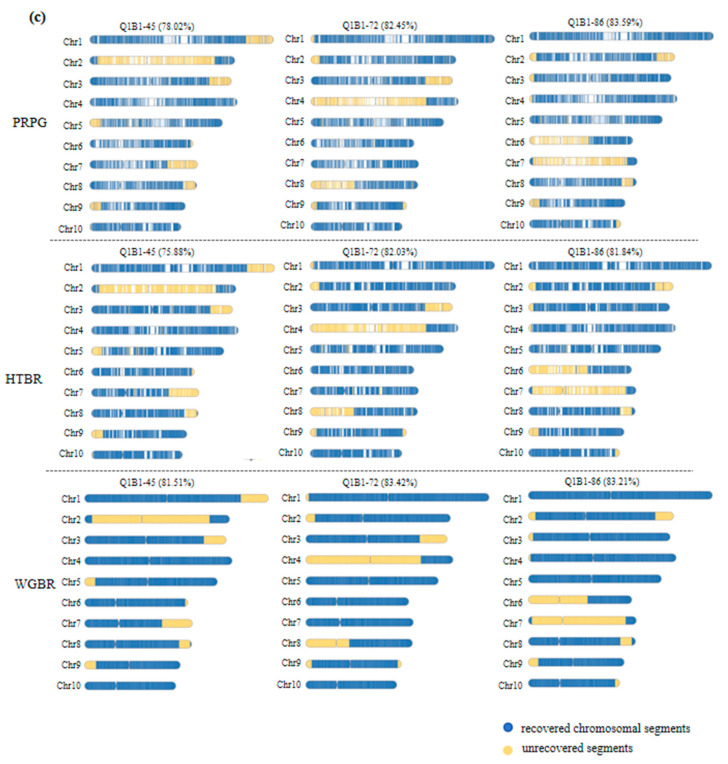
Performance of the G2H strategy versus traditional marker-based background assessment: (**a**) Correlations between HTBR and PRPG (R = 0.98, *p* < 0.001) and between WGBR and PRPG (right, R = 0.95, *p* < 0.001). (**b**) Distribution of BRR in the Q1B2 BC_1_F_1_ population, evaluated by the traditional single-point method (blue) and the G2H strategy (yellow). Each point represents an individual plant. (**c**) Chromosomal segment recovery profiles for the three top-performing individuals ranked by BRR. Segments identical to the RP are shown in blue; donor fragments or heterozygous regions are in yellow. Parentheses indicate the BRR value for each plant. Although these plants had similar overall BRR values, the number, size, and distribution of unrecovered segments (yellow) differ significantly.

To validate the reliability of the G2H assessment system, we calculated the background recovery rate (BRR) of G2H and that of the conventional point marker method in the Q1B1 population, as described in Table S1. We systematically compared its performance against the conventional point marking method in the Q1B1 population using Pearson correlation analysis. As shown in Figure 3a, both methods produced BRR estimates that followed highly consistent trends across individuals, supporting the robustness of the G2H approach. The largest BRR discrepancy between the two strategies was observed in individual Q1B1-58, with a difference of 2.97% (Figure 3b). Importantly, the top three individuals ranked by BRR were identical under both evaluation strategies, further confirming the feasibility of the G2H system in reliably identifying high-recovery progenies.

To evaluate the breeding utility of our method, we analyzed the genetic background of the top three lines with the highest BRR (Figure 3c). While their overall BRR values were comparable, their genomic structures differed markedly. Line Q1B1-86 (highest BRR) harbored numerous small donor fragments, whereas Q1B1-72 featured more fully recovered chromosomal arms.

To quantify these structural differences, we introduced the CFDI. A lower CFDI signifies a more concentrated distribution of unrecovered fragments. For instance, between lines Q1B1-70 (CFDI = 0.42) and Q1B1-78 (CFDI = 0.49) with similar BRR (Table S1), the former had a purer genetic background with fewer, larger fragments. Similarly, among the top lines, Q1B1-72’s lower CFDI (0.12) versus Q1B1-86 (0.24) indicates that its unrecovered segments are more consolidated and thus easier to eliminate in subsequent breeding.

These findings demonstrate that the G2H strategy, augmented by the CFDI, can identify lines with optimal genetic architecture, even those with marginally lower BRR, providing a critical tool for selecting against persistent small fragments and accelerating breeding efficiency.

### 3.3. Evaluation of the High-Generation Backcross Population Background in G2H Strategy

**Figure 4:**
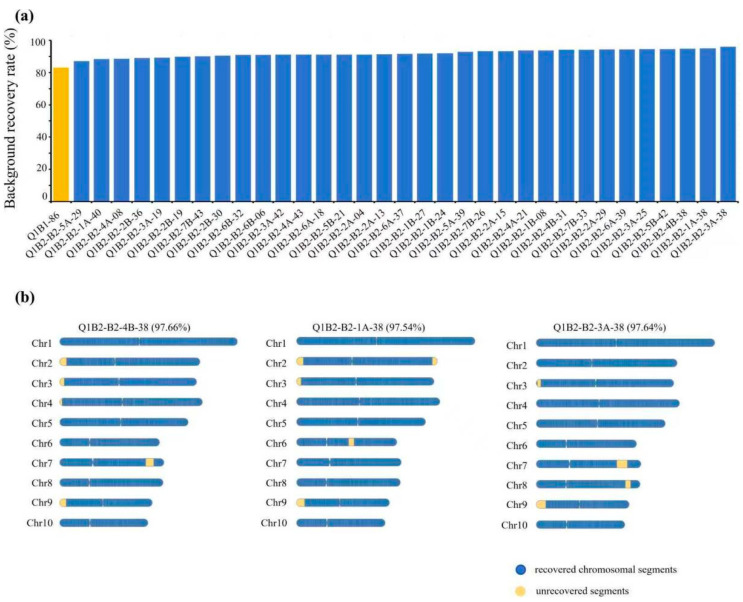
Application of the G2H strategy for genetic background assessment in a BC_2_ population: (**a**) Distribution of BRR across all BC_2_ individuals. (**b**) Chromosomal recovery maps of the three highest-BRR individuals. Recovered (RP-identical) and unrecovered (donor/heterozygous) segments are depicted in blue and yellow, respectively.

To evaluate the performance of the G2H strategy in advanced generations, we analyzed a BC_2_ population derived from the BC_1_ individual Q1B1-86. As shown in Figure 4a, the BRR of the Q1B2 population ranged from 87.09% to 97.64%, with 81.8% (27/33) of individuals exceeding 90% recovery, demonstrating effective genetic background purification through G2H-guided selection.

A detailed structural analysis of the top three BC_2_ individuals ranked by BRR revealed distinct patterns of unrecovered fragments (Figure 4b). All three lines showed elimination of large donor segments on chromosomes 6 and 7, confirming the reliability of G2H in tracking major recombination events. However, variation persisted in the retention of small fragments on chromosomes 2, 3, 4, and 9. Importantly, individuals with slightly lower overall BRR frequently exhibited more favorable fragment distributions, characterized by fewer and more contiguous unrecovered regions, as quantified by the CFDI metric. This structural advantage facilitates more efficient genetic cleanup in subsequent breeding generations.

These findings indicate that the G2H strategy provides not only quantitative BRR estimates but also critical insights into chromosomal architecture. By integrating fragment continuity and distribution into selection criteria, G2H enables the identification of individuals with fewer unrecovered fragments, concentrated distributions, and a more “clean” genetic background. This dual-approach enhances the development of homozygous inbred lines within 1–1.5 years and significantly improves the precision and efficiency of accelerating maize breeding programs.

## 4. Discussion

### 4.1. A Paradigm Shift from Point-Based to Block-Based Genetic Background Assessment

The G2H strategy presented in this study represents a fundamental transition in genetic background assessment by implementing a haplotype block-based framework. By aggregating high-density SNPs into defined haplotype blocks (HTPs), the system advances from discrete locus-based evaluation to continuous chromosomal segment analysis, enabling more accurate reconstruction of recombination events and parental genomic contributions during backcrossing.

This block-based approach leverages the inherent linkage stability within HTPs, where minimal recombination allows for reliable inference of segmental inheritance from limited polymorphic loci. The resulting enhancement in genotyping efficiency and structural resolution provides a more biologically meaningful representation of chromosomal transmission, facilitating the precise identification of recombination breakpoints and quantitative assessment of background recovery.

However, several limitations of the G2H strategy warrant consideration. First, the effectiveness of HTP-based assessment depends on the quality and density of the initial haplotype block map, which may vary across different germplasm and require reconstruction for uncharacterized populations. Second, while the 0.5 threshold for block assignment demonstrated robustness in this study, optimal thresholds may need adjustment when applied to populations with distinct genetic structures or higher heterozygosity. Third, the current implementation requires whole-genome resequencing or high-density SNP genotyping, which may present cost and computational challenges for breeding programs with limited resources.

### 4.2. The CFDI: An Innovative Metric for Addressing Small Fragment Residuals

While BRR quantifies the overall proportion of the recurrent parent genome, it fails to capture the distribution pattern of unrecovered donor fragments—a critical limitation in BC breeding. To address this gap, we introduced the CFDI, a novel metric designed to quantitatively evaluate the dispersion and continuity of residual donor segments across the genome. As demonstrated in our analysis of individuals with comparable BRR values, the CFDI effectively discriminates between favorable and unfavorable fragment distribution patterns. For instance, individual Q1B1-70 (CFDI = 0.42) exhibited fewer, larger unrecovered segments, indicating a more favorable chromosomal structure for rapid background purification compared to Q1B1-78 (CFDI = 0.49), which contained numerous small, dispersed fragments suggestive of a complex recombination history and elevated linkage drag risk.

By complementing BRR with structural information, CFDI enables breeders to select individuals not only based on recovery percentage but also according to chromosomal architecture. This dual-parameter selection strategy significantly enhances the efficiency of parental choice in BC programs, as it prioritizes genotypes with fragment distributions that are more amenable to rapid elimination in subsequent generations.

### 4.3. G2H Technology Advantages and Application Prospects

This study establishes a distinct position in the field by employing block-based HTP markers to analyze the genetic traits of crop populations, differentiating itself from prior research in objectives, methodology, and application focus. Our work concentrates specifically on BC populations, with an emphasis on technical accuracy and practical applicability in breeding. The primary goal is to develop and validate a technology system for precise genetic background assessment. In order to resolve the critical breeding question of “how to accurately evaluate and select optimal individuals in BC populations”, this study resolves the inherent uncertainty in conventional point-marker methods in characterizing true chromosomal recovery.

The G2H strategy developed in this study demonstrates considerable potential for application across a wide range of crop species, owing to its flexible analytical framework and computational foundation. The core component—the HTP block—is readily adaptable, enabling the method to be generalized beyond maize. With the increasing availability of high-quality reference genomes and large-scale resequencing data for major crops such as rice, wheat, and soybean, including recent pan-genome-based haplotype maps in rice [17], the implementation of G2H in these species is becoming increasingly feasible. Moreover, in complex polyploid crops like wheat and cotton, the G2H approach allows for independent HTP partitioning and background evaluation for individual subgenomes (e.g., A, B, D), effectively addressing challenges posed by genomic complexity.

To facilitate effective deployment across crops, we propose establishing standardized crop-specific analysis workflows. Key steps include the following:

Determining optimal HTP block size and density based on species-specific genomic features such as genome size, heterozygosity, and recombination rate;

Recalibrating thresholds for HTP block origin identification using known polymorphism datasets to ensure accuracy across diverse genetic backgrounds;

Embedding core analytical procedures—such as block segmentation, digital noise reduction, and CFDI computation—into programmable bioinformatics pipelines or user-friendly software tools to enable “plug-and-play” functionality across species.

Looking forward, the G2H strategy holds promise for integration with modern breeding platforms.

For instance, when combined with genome-wide selection, HTP blocks can serve as explanatory variables in prediction models, potentially improving accuracy for complex traits. Unlike individual SNPs, HTP blocks potentially capture functional haplotypes more directly. By using HTP blocks as input variables in GS models (e.g., replacing or augmenting SNP matrices), breeders could improve the accuracy of predicting complex, polygenic traits. This approach aligns with the emerging trend of using functional, biologically meaningful units in prediction models. For instance, the genetically regulated components extracted from omics data in the GRAD model share a conceptual similarity with the inheritance-aware nature of HTP blocks. Applying a similar principle, HTP-based GS could be particularly powerful for traits where haplotype diversity and historical recombination are strong determinants of performance.

Second, the G2H approach can concurrently track the introduction of many target loci and evaluate their background purity when combined with gene editing (CRISPR) for polygenic aggregation breeding, allowing for effective synergistic enhancement across numerous traits [18]. For example, when using CRISPR to pyramid multiple disease resistance genes, the G2H system can monitor background purity, concurrently track the recovery of the recurrent parent’s genetic background in each generation following editing, ensuring that the elite line’s agronomic quality is preserved. The G2H system can identify ideal edit carriers, select edited progeny that not only carry the desired edits but also possess the most “clean” genetic background (high BRR and low CFDI), minimizing linkage drag and accelerating the development of a commercial-quality line. In the end, through integration with information systems for breeding management, the G2H strategy will advance BC breeding from its conventional, experience-dependent model to a new paradigm of precision design breeding—one that is completely quantifiable, predictable, and optimizable.

## 5. Conclusions

The G2H strategy establishes an analytical system that transitions from discrete “point markers” to continuous “haplotype blocks” by converting high-density SNP markers into 6163 defined haplotype blocks. This overcomes the inability of conventional methods to resolve true chromosomal structures, providing enhanced chromosomal resolution for BC breeding. The introduction of the CFDI enables, for the first time, quantitative evaluation of the distribution characteristics of unrecovered fragments. This allows breeders to screen ideal individuals with superior genetic structure and easier subsequent purification while maintaining a high background recovery rate, providing crucial decision-making support for accelerating genetic background homogenization. Empirical studies demonstrate that precision selection based on the G2H strategy can yield elite individuals with a background recovery rate exceeding 90% as early as the BC_2_ generation. This shortens the breeding cycle by 2–3 generations compared to conventional methods, providing a reliable technical foundation for an efficient breeding model capable of developing pure inbred lines within 1.5 years.

## Data Availability

Data will be made available upon request.

## Supplementary Materials

The following supporting information can be downloaded at: https://www.mdpi.com/article/10.3390/genes16121480/s1, Table S1: Analysis results of genetic background assessment in population Q1B1; Table S2: Analysis results of genetic background assessment in population Q1B2.

## Abbreviations

BC	Backcross
G2H	Genotype-to-Haplotype
CFDI	Chromosomal Fragment Distribution Index
DP	Donor Parent
RP	Recurrent Parent
MAS	Marker-Assisted Selection
SSR	Simple Sequence Repeat
SNP	Single-Nucleotide Polymorphism
BRR	Background Recovery Rate
HTP	Haplotype Tag Polymorphism
PIC	Polymorphism Information Content
RIL	Recombinant Inbred Line
PRPG	Proportion of Recurrent Parent Genome
HTBR	HTP-based Recurrent Parental Background Recovery Rate
WGBR	Whole-Genome Background Recovery Rate
